# The Influence of Rheumatoid Arthritis and Osteoarthritis on the Occurrence of Arterial Hypertension: An 8-Year Prospective Clinical Observational Cohort Study

**DOI:** 10.3390/jcm12227158

**Published:** 2023-11-18

**Authors:** Dražen Bedeković, Damir Kirner, Ivica Bošnjak, Aleksandar Kibel, Sandra Šarić, Srđan Novak, Višnja Prus

**Affiliations:** 1 Department of Cardiovascular Diseases, Internal Medicine Clinic, University Hospital Osijek, J. Huttlera 4, 31000 Osijek, Croatia; drbedekovic@yahoo.com (D.B.); kirner.damir@gmail.com (D.K.); aleksandar_mf@yahoo.com (A.K.); smakarovic36@gmail.com (S.Š.); 2 Faculty of Medicine Osijek, Department of Internal Medicine, J. J. Strossmayer University, J. Huttlera 4, 31000 Osijek, Croatia; 3 Faculty of Medicine Osijek, Department of Physiology and Immunology, J. J. Strossmayer University, J. Huttlera 4, 31000 Osijek, Croatia; 4 Scientific Centre of Excellence for Personalized Health Care, J. J. Strossmayer University of Osijek, J. Huttlera 4, 31000 Osijek, Croatia; 5 Department of Rheumatology and Clinical Immunology, University Hospital Rijeka, 51000 Rijeka, Croatia; srdan.novak@uniri.hr; 6 Faculty of Medicine Rijeka, Department of Internal Medicine, University of Rijeka, Braće Branchetta 20/1, 51000 Rijeka, Croatia; 7 Department of Rheumatology and Clinical Immunology, Internal Medicine Clinic, University Hospital Osijek J. Huttlera 4, 31000 Osijek, Croatia; prus1@net.hr

**Keywords:** rheumatoid arthritis, osteoarthritis, hypertension, patient outcomes, cardiovascular risk, cardiovascular mortality

## Abstract

Rheumatoid arthritis (RA) increases the risk of cardiovascular mortality and morbidity, including a 50–60% increased risk of cardiovascular disease (CVD). Arterial hypertension (HT) is considered the major contributing risk factor for CVD development in RA patients. In this investigation, we compared the incidence and prevalence of HT between RA and osteoarthritis (OA) and the influence of HT on CVD development in CVD-naive patients in both groups. This was a prospective clinical cohort investigation with an 8-year follow-up period. A total of 201 participants, 124 with RA (investigation group) and 77 with OA (control group), without diagnosed CVD or symptomatic heart failure were included. After selection according to inclusion and exclusion criteria, both groups underwent initial and final visits, and the investigation group underwent annual visits to assess disease activity. Case report forms were completed for each visit. The obtained data were analyzed by a statistician. No difference in the incidence or prevalence of HT was found between the investigation and control groups. No difference in the prevalence of HT was reported between the study groups and age-standardized data from the general population. The investigation group had a higher incidence of CVD than the control group. RA participants with long-term remission had a marginally lower HT prevalence. Although previous studies reported a higher HT prevalence in RA than in OA and the general population, our findings did not support this. The RA group had a higher incidence of CVD, but it is possible that optimal disease control with long-term remission could reduce HT incidence and prevalence while also having beneficial effects on other cardiovascular risk factors (CV) and, consequently, CVD occurrence.

## 1. Introduction

Rheumatoid arthritis (RA) is a chronic autoimmune inflammatory systemic disease that primarily affects the cartilage and underlying bone of small- and medium-sized joints, blood vessels, and various internal organs and shortens patient lifespan by 5–18 years, mainly due to increased cardiovascular (CV) morbidity and mortality [1,2,3,4,5,6,7,8,9,10,11,12,13,14,15,16]. Many large-scale studies have confirmed this hypothesis. A meta-analysis of 24 studies comprising 111,758 patients with 22,927 cardiovascular events reported a 50% increased risk of CV death in patients with RA: 59% and 52% for ischemic heart disease and stroke, respectively [13]. A vicious cycle generated by traditional CV risk factors and persistent chronic inflammation leads to accelerated atherosclerosis, which is a major cause of CV disease (CVD) and increases CV mortality [17]. Arterial hypertension (HT) plays a major role in RA [18,19,20,21,22]. Most studies with a large number of participants and meta-analyses have shown an increased prevalence of HT in RA patients, ranging from 53% to 73%, compared to that in the general population [7,15,18,19,20,23,24,25,26,27,28,29]. An early, large population-based study addressing this issue was conducted by Han et al. in 2006 and provided strong evidence of increased HT prevalence in RA, where the incidence of hypertension was 31% in RA patients compared with 23% in the control population [30]. An increasing trend of HT prevalence (from 24.5% to 37.4%) with RA duration was reported during a 5-year follow-up in a prospective study by Innala et al. [31]. An increased prevalence of HT was also found in RA patients with known CV disease compared with that in RA patients without CV disease [22]. In the general population, obesity is strongly related to hypertension, with a linear relationship between an increase in body mass index (BMI) and systolic, diastolic, and pulse pressures, according to the National Health and Nutrition Examination Survey [32]. Panoulas et al. also reported a significant association between HT and a high BMI and prednisolone use as well as an association of uncontrolled HT with increased BMI and CV events [33]. Studies addressing comorbidities in early RA observed an increased prevalence of HT. Gherghe et al. reported an HT prevalence of 18.2% (15.5% to 21.3%) [20]. A Swedish prospective study with 726 patients reported a significant comorbidity burden at RA onset in 53.2% of the patients, as well as hypertension (27.3%), obstructive pulmonary disease (13.9%), diabetes (8.0%), hypothyroidism (6.3%), and malignancy (5.0%). At a 5-year follow-up, 41.0% of patients developed at least one new comorbidity: hypertension (15.1%), malignancy (7.6%), stroke/transient ischemic accident (5.1%), myocardial infarction (4.3%), and osteoporosis (3.7%), with high disease activity being associated with the occurrence of a new comorbidity [26].

However, data from the Finnish nationwide register of 7209 patients with RA reported a higher prevalence of coronary heart disease, but not hypertension, at the time of RA diagnosis, with the exception of rheumatoid factor-negative patients who had increased hypertension prevalence [34]. A 2010 meta-analysis by Boyer et al. of 15 case-control studies with 2956 RA patients and 3713 controls did not find significant differences in HT and dyslipidemia prevalence between groups [35]. A British population-based study also did not report a significantly increased HT prevalence in RA patients compared with that in the general population, but the HT incidence was increased [25]. Another multicentric study with 977 participants, published in 2012 by Vergles et al., did not show a significant difference in HT prevalence between RA and osteoarthritis (OA) patients [36].

The cardiovascular risk in patients with RA and OA is similar or it is slightly lower in patients with OA [7,12,13,15,37,38,39,40,41,42,43]. The CV risk for OA is significant owing to the higher prevalence of traditional risk factors than that for RA and older age at the average time of OA diagnosis [42,43] The risk is further increased by the use of drugs to treat OA, primarily non-steroidal anti-inflammatory drugs [36]. The risk of CVD for OA patients is estimated to be 55%, and two studies on the prevalence of HT reported a prevalence of 40% and 75% [42,43,44].

An increasing number of studies highlight the problem of the suboptimal diagnosis and treatment of HT in RA patients, especially in older patients and in those with high CV risk [17,18,27,28,45,46,47]. However, a similar situation is commonly observed in the general population [48]. Among a group of RA patients, approximately 22% were unaware of hypertension, and 67.2% were treated with less than half (48.6%) achieving the treatment goals [27]. The use of glucocorticoids and non-steroidal anti-inflammatory drugs increases the risk of HT in RA patients [43,45].

We compared the incidence and prevalence of HT in RA and OA patients and the possible association between CVD occurrence in patients without known CVD in both groups during 8 years of follow-up. The results were also compared with available data from the general population.

## 2. Materials and Methods

This prospective observational cohort study included 201 participants, 124 with RA (investigation group) and 77 with OA (control group), recruited from a rheumatology center at University Hospital Osijek. All RA and OA diagnoses were made by two senior rheumatologists with over 30 years of experience in the field of rheumatology. During the selection process, we manually searched the hospital database and included all eligible patients according to the inclusion and exclusion criteria outlined below. The inclusion period lasted for 1 year. The observational investigation period lasted for an average of 8 years and 4 months ± 3 months, from 2008/9 to 2016/17.

The inclusion criteria for the investigation were:

1. Diagnosis of RA and OA by a rheumatologist according to the 1987 American College of Rheumatology (ACR) revised classification criteria [3].
2. Signed informed consent to participate in the investigation.
3. Registered residence/permanent residence in one of the five counties in Eastern Croatia continuously during the investigation.

The exclusion criteria for the investigation were:

1. Non-fulfillment of inclusion criteria.
2. Rejection of further participation during the investigation period.
3. Diagnosed of CVD or symptomatic heart failure (HF); (any type of myocardial infarction or angina pectoris, significant coronary disease proven on diagnostic tests, symptomatic or asymptomatic ischemic cardiomyopathy, stroke or transient ischemic attack, acute or chronic dissection or aneurysm of the aorta, penetrating aortic ulcer or intramural hematoma, symptomatic or asymptomatic peripheral arterial disease, or suspected CVD based on recorded or detected signs, symptoms, or tests).
4. Permanent cessation of residence in one of the five counties of Eastern Croatia during the investigation period and migration within the region were not exclusion criteria.

Clinical and laboratory methods included a detailed analysis of medical history; the completion of a comprehensive case report form (CRF), a questionnaire written in Croatian for RA and OA; a physical examination with the measurement of blood pressure three times at 10 min intervals (average recorded as the final result), waist and hip circumference, and body weight and height; assessment of general health and pain intensity using visual analog scales (VAS); completion of a disease activity score 28-C-reactive protein (DAS28-CRP), form (RA participants) [49], arthritis severity index for OA of the hand form (OA participants) [50], Lequesene index for OA of the hip form (OA participants) [50], Lequesene index for OA of the knee (OA participants) [50], and the Croatian translation of the Health Assessment Questionnaire (HAQ) (RA and OA participants) [51]; recording a 12-channel electrocardiogram; and collection of venous blood for laboratory analysis of the following parameters: erythrocyte sedimentation rate and C-reactive protein (CRP), total cholesterol, high-density lipoprotein (HDL), low-density lipoprotein (LDL), triglycerides, creatinine, plasma glucose, and glycosylated hemoglobin (HbA1c) levels. An oral glucose load test was performed if indicated. Levels of rheumatoid factor (at the initial and final visits) and anti-citrulline antibodies (at the final visit only) were determined in participants with RA. All CRFs were completed, and all examinations were performed by an investigating doctor; the nurse only collected blood samples.

If, at any visit, an average blood pressure above 140/90 mmHg was detected in non-HT participants, the participant underwent intensive blood pressure monitoring for at least 1 month to confirm or exclude hypertension.

All participants from the investigation and control groups attended their first and final visits (if available), and participants from the investigation group attended annual visits to assess RA activity. Participants and their families were instructed to report any changes in their health status, including the occurrence of CVD or new CVD risk factors, any other disease, surgery, or RA flare (a list was provided), by telephone, e-mail, or personal visits. The reason for any unavailability of participants during the investigation was recorded on a special form. If the participant died, the cause of death and comorbidities were recorded, as well as the known cardiovascular risk factors diagnosed before death. Data were obtained from autopsies, coroners’ reports, reports from the Croatian Institute for Public Health, hospitals, and primary care physician records.

All CRFs are available in the Supplementary Material.

### Statistical Methods

Descriptive statistics were used to describe and summarize the data. Inferential statistics were used to test the hypotheses. An independent samples t-test was employed to check the significance of mean differences. Depending on Levene’s test results, a t-test assuming equal or unequal variance was used to determine statistical significance between groups. A chi-square test of independence was used to analyze the relationships between the qualitative variables. Fisher’s exact test was used when the assumptions for the chi-square test were not met. The level of significance was set at *p* < 0.05, whereas 0.05 < *p* < 0.10 was considered a tendency (marginally significant or trend).

## 3. Results

### 3.1. Comparison of Investigation and Control Groups

Of the 201 participants who were included in the investigation 2008/9, 124 with RA and 77 with OA, 137 had finished the investigation in 2016/17, including 82 with RA and 55 with OA. The reasons for not completing the investigation were as follows: 58 participants (28.9%) died during the investigation: 41 (33.1%) with RA and 17 (22.9%) with OA; 29 (70.7%) of those with RA and 10 (58%) of those with OA died of CVD; and 6 refused to participate or migrated outside the country (1 had RA, and 5 had OA).

At the initial visit, the investigation (RA) group included 25 men and 99 women, and the control (OA) group included 11 men and 66 women. The average age was 59.78 years (37–81) in the RA group and 64.23 years (27–80) in the OA group, OA participants were significantly older (*p* = 0.004). The average disease duration was 12.2 years for the RA group and 5.64 years for the OA group. Of the included RA participants, 82.3% had a positive rheumatoid factor, with an average value of 337.14 kIU/L (range 4–560 kIU/L). The disease activity for RA assessed by DAS28-CRP was an average of 4.94 (standard deviation 1.41, range 1.46–8.08), which suggests moderate disease activity.

At the final visit in 2016/17, the average age of the 137 remaining participants was 65.5 years in the RA group and 71.2 years in the OA group. Of the 82 RA participants who completed the investigation, 16 were men and 66 women, and of the 55 OA participants, 10 were men and 45 women. Of the remaining RA participants, 90.2% had a positive rheumatoid factor, and 80.5% had a positive anti-CCP antibody, with an average value of 347.84 U/mL (range 0.8–2684 U/mL). The disease activity (RA) assessed using DAS28-CRP averaged 4.08 (standard deviation 1.12, range 1.38–6.34), which reflected moderate disease activity. A longitudinal comparison of participants available at the final visit showed a significant decrease in disease activity, but activity remained moderate on average (4.85 vs. 4.08, *t* = 4.78, *p* = 0.001).

The cumulative (alive + deceased) incidence of CVD was 43.9% (55/123 participants) in the RA group and 37.5% (27/72 participants) in the OA group, without a significant difference (*p* = 0.381). CVD incidence among alive participants was 31.7% and 30.9% for RA and OA, respectively, without a significant difference (*p* = 0.912).

The leading cause of death in both groups was CVD (heart failure, stroke, and myocardial infarction). Participants with RA had significantly shorter lifespans (71.59 years for RA vs. 76.94 years for OA; *p* = 0.039).

The use of glucocorticoids in the RA group was continuously high during the investigation (74.2% and 76.8% at the initial and final visits, respectively; *p* = 0.332). The prevalence of painkiller use (non-steroidal anti-inflammatory drugs (NSAIDs), and non-opioid and opioid analgesics) was continuously high in both groups. The usage of any painkillers at the initial visit was 87.1% in RA and 90.9% in OA (*p* = 0.41), while at the final visit, usage was 95.1% for RA and 89.1% for OA (*p* = 0.55). For NSAIDs, the usage at initial visit was 55.1% for RA and 63.9% for OA (marginally significant difference; *p* = 0.051), while at the final visit, it was 75.4% for RA and 60% for OA (*p* = 0.17). The prevalence of classical disease-modifying antirheumatic drugs (DMARDs) was 86.3% at the initial visit and 80.5% at the final visit (no significant difference, *p* = 0.27). The DMARDs used at the initial visit were methotrexate (69 participants), leflunomide (17 participants), and sulfasalazine (65 participants). The DMARDs used at the final visit were hydroxychloroquine (10 participants), methotrexate (56 participants), leflunomide (3 participants), and sulfasalazine (29 participants). Biologic agents were used by only fifteen participants during the study, and three of them discontinued the treatment (two died, one adverse effect). The prevalence of biologic agent usage was 2.24% (3 participants) at the initial visit and 14.6% (12 participants) at the final visit, which represents a significant increase in biologic agent use (*p* = 0.001). The types of biologic agents used at the initial visit were etanercept (two participants) and adalimumab (one participant). The biologic agents used at the final visit were adalimumab (six participants), infliximab (two participants), etanercept (two participants), certolizumab (one participant), and tofacitinib (one participant).

During the investigation, a continuous comparison of the prevalence of HT between the investigation group (RA) and control group (OA) did not show a statistically significant difference in prevalence (2008/9 RA: 62.68.8%, OA: 68.8% (*p* = 0.332); 2016/17 RA: 73.2%, OA: 80% (*p* = 0.359). There was also no evidence of increased prevalence at the end of the investigation cumulatively for the sum of living and deceased participants between the groups (RA 75.6%, OA 77.8%, (*p* = 0.731). Table 1 and Table 2 show the general characteristics of the participants at the initial and final visits, including the number of CV risk factors and antihypertensive used. 

The types of antihypertensive drugs used at the beginning of the investigation for the treatment of 77 participants with hypertension in the RA group were as follows: beta blockers in 15 participants, angiotensin-converting enzyme (ACE) inhibitors in 35 participants, calcium channel blockers in 22 participants, AT1 blockers in 1 participant, diuretics in 16 participants, and other antihypertensive drugs in 6 participants. The types of antihypertensive drugs used at the beginning of the investigation for the treatment of 53 participants with hypertension in the OA group were as follows: beta blockers in 16 participants, ACE inhibitors in 38 participants, calcium channel blockers in 19 participants, diuretics in 24 participants, and other antihypertensive medications in 4 participants. The types of antihypertensive drugs used at the end of the investigation for the treatment of 60 participants with hypertension in the RA group were as follows: beta blockers in 15 participants, alpha blockers in 1 participant, ACE inhibitors in 37 participants, calcium channel blockers in 24 participants, AT1 blockers in 1 participant, diuretics in 22 participants, and other antihypertensive drugs in 1 participant. The types of antihypertensive drugs at the end of the investigation for the treatment of 44 participants with hypertension in the OA group were as follows: beta blockers in 15 participants, ACE inhibitors in 24 participants, calcium channel blockers in 22 participants, AT1 blockers in 9 participants, diuretics in 15 participants, and other antihypertensive medications in 2 participants.

### 3.2. Influence of Age on Risk Factors and Incidence of CVD in RA and OA

In the subgroup analysis of the influence of age on the participants, the investigation and control groups were further divided into two subgroups: younger and older than 65 years of age. In the investigation (RA) group, participants under 65 years achieved significant results compared with those over 65 years: shorter duration of HT (*p* = 0.012), lower average systolic (*p* = 0.001) but not diastolic pressure (*p* = 0.272), generally better health condition (GH assessment) (*p* = 0.001), lower DAS28 and HAQ (*p* = 0.001) scores, lower chronic pain (VAS) (*p* = 0.02), lower prevalence of hypertension (*p* = 0.001) but higher prevalence of current (*p* = 0.003) and former smokers (*p* = 0.009), lower prevalence of heart disease in general (*p* = 0.01), lower incidence of CVD (*p* = 0.018), lower prevalence of metabolic syndrome (*p* = 0.004), and marginally lower incidence of heart disease in general (*p* = 0.06) and prevalence of antihypertensive drug usage (*p* = 0.069). In the control (OA) group, for participants under 65 years compared to those over 65 years, we highlighted lower HAQ (*p* = 0.002) and HDL values (*p* = 0.001), a significantly increased prevalence of current (*p* = 0.006) and former smokers (*p* = 0.002), and a trend of shorter duration of hypertension (0.086), a lower number of CVD risk factors (*p* = 0.083), and a lower incidence of myocardial infarction (0.069).

In a comparison of equivalent subgroups of the investigation (RA) and control (OA) groups for those under 65 years, the results showed a longer disease duration with RA (*p* = 0.001), lower systolic pressure (*p* = 0.033) and BMI (*p* = 0.021) and higher HDL values (*p* = 0.001) and a lower prevalence of general heart disease (0.023) in the RA group, while a marginally significantly shorter duration of hypertension (*p* = 0.068) and increased incidences of CVD (*p* = 0.09) and stroke (0.054) were also shown for RA compared to those for OA but were not statistically significant. In a comparison of subgroups for those over 65 years, RA showed a significantly longer disease duration (*p* = 0.001); higher values of systolic pressure (*p* = 0.038), CRP (*p* = 0.002), and sedimentation (*p* = 0.014); a higher prevalence of ex-smokers (0.015); and a greater number of risk factors for CVD compared to OA (*p* = 0.018), while these trends were present in the RA group: higher HAQ (*p* = 0.09), lower overall health status (GH) (*p* = 0.09), prevalence of current smokers (*p* = 0.09), and lower incidence of heart failure (*p* = 0.094) and CVD (*p* = 0.079) in relation to OA. There was no difference in the incidence or prevalence of hypertension between the two groups (*p* = 0.481 for both).

### 3.3. The Influence of Optimal Inflammatory Disease Control (RA)

The control of chronic inflammation within the investigation group (RA) was analyzed, wherein the subjects were divided into two groups: good control (remission) and unsatisfactory control of inflammation. After the first visit, participants from the research group had regular visits/examinations every 12 to 18 months, where disease activity (DAS28-CRP score) was monitored, clinical disease activity was assessed, inflammatory laboratory parameters (CRP, sedimentation, blood count) were analyzed, and all relevant events related to RA were recorded as well as all newly diagnosed diseases. The criterion for the remission/low activity group was DAS28-CRP < 3.2 for at least 60% of the investigation time, provided that the increase in inflammatory parameters was caused by RA activity. Sixteen participants achieved long-term remission, whereas fifty-three had unsatisfactory inflammation control, and thirteen participants missed more than two annual visits and were not analyzed in this subgroup. Participants with long-term remission were shown to have statistically significantly shorter duration of RA (0.001); lower values of waist/hip ratio (*p* = 0.032), HAQ (*p* = 0.006), and VAS pain assessment (*p* = 0.021); a better assessment of the overall health status (*p* = 0.028); higher cholesterol (0.037) and HDL (*p* = 0.043) but not LDL (*p* = 0.51) values; lower blood glucoses values (*p* = 0.003), HbA1c (*p* = 0.005), and use of glucocorticoids (*p* = 0.001); a trend towards a lower prevalence of diabetes (*p* = 0.058); a lower incidence of heart failure (*p* = 0.056); and a lower number of risk factors for CVD (*p* = 0.034). In addition, the optimal control of RA inflammation showed trends toward lower BMI values (*p* = 0.058) and a lower prevalence of HT (0.067). Table 3 and Table 4 show the parameters measured at the initial and final visits, respectively.

### 3.4. The Influence of Disease Duration

Subgroup analysis according to disease duration (<15 years, 15–25 years, and >25 years) showed a significantly higher prevalence of current and ex-smokers in RA and higher HDL levels in the <15 years group compared to the matched OA group. No significant differences were found for other cardiovascular risk factors and CVD in any matched subgroups.

## 4. Discussion

The main results of this investigation suggest that there is no significant difference in the prevalence of HT between participants with RA and those with OA. The prevalence of HT did not differ significantly between the groups throughout the study period. The incidence of HT was similar between the study groups. Regarding trends within the investigation groups, HT prevalence was significantly increased in each investigation group at the end of the investigation compared to that at the start of the investigation, which might be explained by the increase in the age of the participants but not by the disease (RA or OA) duration, as evident in the subgroup analyses.

The main strength of this study was its prospective design with a long follow-up period (8 years). Previous studies have investigated the prevalence of HT in RA; however, the results have been contradictory. Han et al. found strong evidence of increased HT prevalence in RA, with an incidence of hypertension of 31% compared with 23% in the control population [30]. Innala et al. reported an increasing trend in HT prevalence with RA duration during a 5-year follow-up period. [31]. RA patients with known CVD also showed an increased prevalence of HT compared with RA patients without CVD in a Greek cohort study by Serelis et al. [22]. However, some studies have failed to show a higher prevalence of HT in RA than in control groups. Examples of such studies include the previously mentioned multicentric investigation by Vergles et al. in 2012, which did not show a significant difference in HT prevalence between RA and OA patients [36]; a British population-based study [25]; a meta-analysis of 15 case-control studies published in 2010 by Boyer et al. [35]; and a Finnish nationwide register of 7209 RA patients, which reported a higher prevalence of coronary heart disease but not hypertension at the time of RA diagnosis, with the exception of rheumatoid factor-negative patients who had increased hypertension prevalence [34]. Therefore, the present study aimed to prospectively investigate the prevalence of HT in participants with RA and OA, using a protracted follow-up period, as a means to better detect any differences in HT prevalence that may develop over time in participants with inflammatory diseases who might be at an increased risk of HT because of the inflammatory process, which is a known risk factor that leads to vascular changes [17].

By comparing our investigation results with the Croatian study (EH-UH) [52] and comparing the HT prevalence in the population (crude prevalence was 37.5%) with the prevalence of HT for RA and OA, the crude results showed a higher prevalence of HT in both groups during the investigation period (*p* < 0.0001). However, when the prevalence of HT was standardized according to age and compared with the HT prevalence in the Croatian population, it was not statistically significantly different. This is in agreement with the investigation by Vergles et al. [36].

Interestingly, the results suggest that HT prevalence is not higher in patients with RA than in those with OA. Considering the inflammatory and autoimmune pathogenesis of RA, it is worth noting that, in a Colombian cross-sectional study of patients with RA and OA, there were no reported differences in the markers of vascular aging (as measured by pulse wave velocity/arterial stiffness) between patients with RA who had low levels of disease activity and those with OA who had poor metabolic control [53]. Since it has been shown that there is a correlation between arterial blood pressure and pulse wave velocity [54,55], there seems to be concordance between the results of the cross-sectional study investigating vascular age in RA and OA and our investigation of the prevalence of HT in participants with these diseases. In a large study by Wolfe et al. on 11,572 patients (9093 with RA, 2479 with OA), RA was shown to be associated with an increased risk of cardiovascular and/or cerebrovascular disease due to myocardial infarction, congestive heart failure, and probably cerebrovascular accidents [56]. The Korea National Health and Nutrition Examination Survey conducted from 2010 to 2012 found that, among other factors, patients with RA had more comorbidities, including hypertension [57]. Such apparent discrepancies suggest that there are still unknown factors in our understanding of the interplay between HT, CVD risk, and RA/OA.

At the initial visit, BMI values were significantly lower in the RA group when compared to the OA group, but this difference disappeared during the investigation period. Average values remained continuously above normal. Mean BMI was significantly higher in people with hypertension than those without for both RA and OA groups at the initial visit (*p* = 0.05 and *p* = 0.001, respectively), and at the final visit (*p* = 0.037 and *p* = 0.027, respectively). HT was associated with BMI > 25 at the initial visit for both groups (*p* = 0.01 for RA and OA), but at the final visit, this association remained for RA only (*p* = 0.02 for RA, *p* = 0.124 for OA). According to published studies, obesity is a known CV risk factor, as is hypertension, which is in concordance with our results. RA and OA have a higher prevalence than in the general population, more pronounced in OA patients, and RA increases CV morbidity, mortality, and disease severity and decreases chances to achieve and sustain remission compared to normal-weight patients [25,32,58,59,60,61]. 

Furthermore, our investigation demonstrated a statistically significant association between the prevalence of HT and the incidence of CVD in the RA group for living participants and the sum of living and deceased participants (*p* = 0.033 and *p* = 0.009, respectively) but not in the OA group (*p* = 0.471 and *p* = 1, respectively). An increase in the number of CVD risk factors was observed in the RA group at the end of the study period. Dessein et al. found an excess cardiovascular risk in RA patients compared to that in OA patients, primarily including the presence of decreased insulin sensitivity and HDL cholesterol in RA patients [61]. These findings are in concordance with the trend observed in CVD risk in the RA group, as observed in our investigation.

This study has some limitations. The investigation included a comparably smaller participant group than some previous epidemiological studies investigating the association between HT and systemic diseases. However, a strength is the long follow-up of the prospectively included participants, which in our opinion outweighs the limitations. An additional advantage of our investigation compared to epidemiological studies is the design that included only CVD and symptomatic heart failure-naïve participants, who were strictly followed up to obtain accurate data about their health status and minimize possible bias.

By choosing patients with OA as controls, we tried to minimize the influence of two confounding factors: NSAIDs/analgesic usage and limitation in physical activity (HAQ). The initial use of NSAIDs (but not cumulative use of non-opioid or opioid analgesics) was slightly higher in the OA group, and HAQ was higher in the RA group. However, by the end of the investigation, there was no significant difference between the groups. Baseline characteristics and differences between RA and OA participants, including a higher prevalence of females in both groups, higher age in the OA group, longer disease duration for RA, a higher proportion of smokers in RA, and higher BMI in OA, are consistent with reported epidemiological and clinical features of the disease [62]. The level of disease activity (RA) remained moderately high with a tendency to improve, probably owing to the introduction of more effective medications. To further minimize the effect of confounders, we performed subgroup analyses based on disease duration and age, where the matched investigation and control groups showed no differences in the incidence and prevalence of HT.

The aggravating influence on HT and CVD risk of some medications used for RA (but not OA) is well known. The prevalence of long-term glucocorticoid use was continuously high during the study and had a proportionally constant effect. Leflunomide, which can promote HT development [63,64], was used by a small proportion of participants, and its use declined during the study (*p* = 0.017). We found no association between leflunomide usage and HT prevalence (*p* = 0.81 and *p* = 1 for initial and final visits, respectively). Etanercept was used by two participants. The ATTACH trial found that anti-TNF therapy increased mortality or worsened heart failure in patients with moderate to severe ischemic chronic heart failure, but the RENAISSANCE and RECOVER clinical trials did not confirm this for enanercept [65,66]. Certolizumab also increases the incidence of hypertension [67] but was used by only one participant, so its effect on the investigation results is not significant. Tofacitinib, used by one participant, can increase cholesterol, HDL, and LDL levels, but it has an overall beneficial effect on mortality and incidence of cardiovascular events [68].

There is always the possibility of confounding factors influencing a complex clinical entity, such as hypertension, which has a multifactorial etiopathogenesis (and not all of the mechanisms are fully elucidated); therefore, there is a possibility that such factors skew the results. Lastly, some participants were lost to follow-up, but this was mainly due to the death of the participants, and only a very small number of participants were lost because of other reasons.

In conclusion, the present investigation did not find a statistically significant difference in HT incidence and prevalence between RA and OA participants but discovered a significant association between the prevalence of HT and the incidence of CVD in the RA group, although the results of this investigation do not support the conclusions of most previous studies. A higher incidence of CVD in RA participants is expected and has been confirmed by numerous studies; however, there is a possibility that optimal disease control with long-term remission could reduce HT prevalence and have a beneficial effect on other cardiovascular risk factors and, subsequently, CVD occurrence. The main strength of this investigation is the long follow-up period, which might contribute to a better general picture of the interplay between HT and other risk factors of CVD in systemic diseases; however, further research is necessary, especially focusing on the mechanisms of RA-induced effects on the cardiovascular system, blood pressure regulation, and consequently, HT.

## Figures and Tables

**Table 1:** General participant data at the initial visit 2008/9.

Prevalence (%)	Rheumatoid Arthritis Group—Visit 2008/9. (N = 124)	Osteoarthritis Group—Visit 2008/9. (N = 77)
male sex	20.2	14.3
female sex	79.8	85.7
use of glucocorticoids currently	74.2	
arterial hypertension	62.1	68.8
smoking cigarettes ever	46.8	27.3
smoking cigarettes active	21.8	14.3
number of risk factors of coronary disease 0	5.6	3.9
number of risk factors of coronary disease 1	26.6	29.9
number of risk factors of coronary disease 2	47.6	49.4
number of risk factors of coronary disease 3	20.2	15.6
number of risk factors of coronary disease 4	0	1.3
heart diseases total/non-ischemia for 2008 *	16.1	16.1
use of antihypertensive medicines (only subjects with hypertension)	71.4 (N = 77)	86.8 (N = 46)
number of antihypertensive 0	28.5 (N = 77)	13.2 (N = 46)
number of antihypertensive 1	32.5 (N = 77)	22.64 (N = 46)
number of antihypertensive 2	29.9 (N = 77)	32.1 (N = 46)
number of antihypertensive 3	5.2 (N = 77)	24.53 (N = 46)
number of antihypertensive 4	3.9 (N = 77)	7.55 (N = 46)
number of antihypertensive 5	0 (N = 77)	0 (N = 46)

* Diseases not included in the exclusion criteria: mild valvular disease, hypertonic heart, nonsignificant congenital disease.

**Table 2:** General participant data at the final visit 2016/17.

Prevalence (%)	Rheumatoid Arthritis Group—Visit 20016/17. (N = 82)	Osteoarthritis Group 20016/17. (N = 55)
male sex	19.5	18.2
female sex	80.5	81.8
use of glucocorticoids currently	76.8	
arterial hypertension	73.2	80
smoking cigarettes ever	52.4	25.5
smoking cigarettes active	29.3	10.9
number of risk factors of coronary disease 0	2.4	1.8
number of risk factors of coronary disease 1	9.8	29.1
number of risk factors of coronary disease 2	52.4	38.2
number of risk factors of coronary disease 3	29.3	27.3
number of risk factors of coronary disease 4	6.1	3.6
heart diseases total/all for 2016	37.8	49.1
use of antihypertensive medicines (only subjects with hypertension)	91.7 N = (60)	95.5 (N = 42)
number of antihypertensive 0	8.3 N = (60)	4.5 (N = 42)
number of antihypertensive 1	36.7 N = (60)	29.5 (N = 42)
number of antihypertensive 2	30 N = (60)	40.9 (N = 42)
number of antihypertensive 3	16.7 N = (60)	11.4 (N = 42)
number of antihypertensive 4	6.7 N = (60)	13.6 (N = 42)
number of antihypertensive 5	1.7 N = (60)	0 (N = 42)
arterial hypertension (alive + deceased)	75.6 (N = 123)	77.8 (N = 72)

**Table 3:** Measured parameters at the initial visit.

Group		N	Mean	Std. Deviation	Std. Error Mean	Statistically Significant Difference	*t*	*p*
Systolic BP 2008	Rheumatoid arthritis	124	136.96	17.47	1.57	No—marginal, RA has higher	1.735	0.084
	Osteoarthritis	77	133.17	13.35	1.52			
Diastolic BP 2008	Rheumatoid arthritis	124	84.30	9.19	0.83	No—marginal, RA has higher	1.961	0.051
	Osteoarthritis	77	81.82	7.89	0.90			
BMI 2008	Rheumatoid arthritis	124	28.00	5.88	0.53	Yes—RA has lower	−2.58	0.011
	Osteoarthritis	77	30.07	4.96	0.57			
Waist- hip ratio 2008	Rheumatoid arthritis	124	0.91	0.10	0.01	No	0.638	0.524
	Osteoarthritis	77	0.90	0.08	0.01			
HAQ 2008	Rheumatoid arthritis	124	1.65	0.93	0.08	Yes—RA has higher	2.438	0.016
	Osteoarthritis	77	1.40	0.55	0.06			
VAS 2008	Rheumatoid arthritis	124	6.02	2.46	0.22	No	0.712	0.477
	Osteoarthritis	77	5.77	2.34	0.27			
GH 2008	Rheumatoid arthritis	124	43.42	25.39	2.28	No	−0.237	0.813
	Osteoarthritis	77	44.16	18.47	2.10			
ESR 2008	Rheumatoid arthritis	122	35.34	25.06	2.27	Yes—RA has higher	5.532	0.001
	Osteoarthritis	74	19.70	14.49	1.68			
Total cholesterol 2008	Rheumatoid arthritis	124	5.85	1.24	0.11	No	−0.39	0.697
	Osteoarthritis	77	5.93	1.38	0.16			
HDL 2008	Rheumatoid arthritis	123	1.53	0.46	0.04	No	0.395	0.693
	Osteoarthritis	75	1.48	1.40	0.16			
Triglycerides 2008	Rheumatoid arthritis	124	1.65	0.70	0.06	Yes—RA has lower	−3.46	0.001
	Osteoarthritis	77	2.22	1.34	0.15			
Creatinine 2008	Rheumatoid arthritis	124	79.03	19.95	1.79	No	0.971	0.408
	Osteoarthritis	75	76.69	18.16	2.10			
Blood glucose 2008	Rheumatoid arthritis	124	5.68	1.89	0.17	No	−0.976	0.33
	Osteoarthritis	77	5.96	2.22	0.25			

**Table 4:** Measured parameters at the final visit.

Group		N	Mean	Std. Deviation	Std. Error Mean	Statistically Significant Difference	t	*p*
Systolic BP 2016	Rheumatoid arthritis	82	131.84	18.01	1.99	No	−0.296	0.758
	Osteoarthritis	55	132.71	14.94	2.01			
Diastolic BP 2016	Rheumatoid arthritis	82	80.82	9.41	1.04	No	0.25	0.803
	Osteoarthritis	55	80.40	9.84	1.33			
BMI 2016	Rheumatoid arthritis	82	27.90	5.42	0.60	No	−1.482	0.141
	Osteoarthritis	55	29.27	5.16	0.70			
Waist- hip ratio 2016	Rheumatoid arthritis	82	0.91	0.08	0.01	No	−1.009	0.315
	Osteoarthritis	55	0.92	0.09	0.01			
HAQ 2016	Rheumatoid arthritis	82	1.60	0.87	0.10	No	0.444	0.658
	Osteoarthritis	55	1.53	0.76	0.10			
VAS 2016	Rheumatoid arthritis	80	4.96	2.38	0.27	No	−0.131	0.896
	Osteoarthritis	55	5.02	2.50	0.34			
GH 2016	Rheumatoid arthritis	80	41.54	22.17	2.48	No	0.285	0.776
	Osteoarthritis	55	40.38	24.51	3.30			
ESR 2016	Rheumatoid arthritis	82	28.61	17.63	1.95	No—marginal, RA has higher	1.675	0.096
	Osteoarthritis	55	23.33	18.79	2.53			
Total cholesterol 2017	Rheumatoid arthritis	82	5.86	1.00	0.11	No	−0.171	0.864
	Osteoarthritis	55	5.90	1.31	0.18			
HDL 2016	Rheumatoid arthritis	82	1.53	0.37	0.04	Yes—RA has higher	3.145	0.002
	Osteoarthritis	55	1.34	0.35	0.05			
Triglycerides 2016	Rheumatoid arthritis	82	1.62	0.78	0.09	No	−1522	0.13
	Osteoarthritis	55	1.83	0.80	0.11			
Creatinine 2016	Rheumatoid arthritis	82	72.01	21.36	2.36	No	−0.807	0.421
	Osteoarthritis	55	75.02	21.40	2.89			
Blood glucose 2016	Rheumatoid arthritis	82	6.39	3.16	0.35	No	0.214	0.831
	Osteoarthritis	55	6.28	2.49	0.34			
HbA1c%	Rheumatoid arthritis	82	5.97	1.38	0.15	No	1.056	0.293
	Osteoarthritis	55	5.71	1.42	0.19			

## Data Availability

The datasets used or analyzed in this investigation are available from the corresponding author upon request. All source documents (preselection documents, initial visit questionnaire and test results, annual visit questionnaire and test results, final visit questionnaire and test results) are in paper form, written in Croatian, and consist of participant identification data. If requested, all participant identification data have to be removed (name, surname, social security number, personal identification number), as well as all the information in the datasets that consist of participant identification data and is written in Croatian.

## Supplementary Materials

The following supporting information can be downloaded at: https://www.mdpi.com/article/10.3390/jcm12227158/s1, S1: CRF RA initial visit, S2: CRF OA initial visit, S3: CRF RA annual visit, S4: CRF RA final visit, S5: CRF RA final visit—unavailable, S6: CFR OA final visit, S7: CRF OA final visit- unavailable, S8: HAQ form, S9: Lequesene hip Osteoarthrits form, S10: Lequesene knee Osteoarthrits form, S11: Osteoarthrits fist index form, and S12: DAS28 CRP form.
