# Supplementary material for: The Influence of Rheumatoid Arthritis and Osteoarthritis on the Occurrence of Arterial Hypertension: An 8-Year Prospective Clinical Observational Cohort Study
Source: J Clin Med. 2023 Nov 18;12(22):7158. doi: 10.3390/jcm12227158 (PMC10672072; doi:10.3390/jcm12227158)
Supplement: Supplementary file 1 [file jcm-12-07158-s001.zip › S12 DAS 28 CRP score form.pdf]

DAS28 - bodovi

Prezime: ....

Ime: ....

Datum rođenja: ....

Datum pregleda: ....

ZGLOBOVI BOLNI NA DODIR
(br. zbd.):

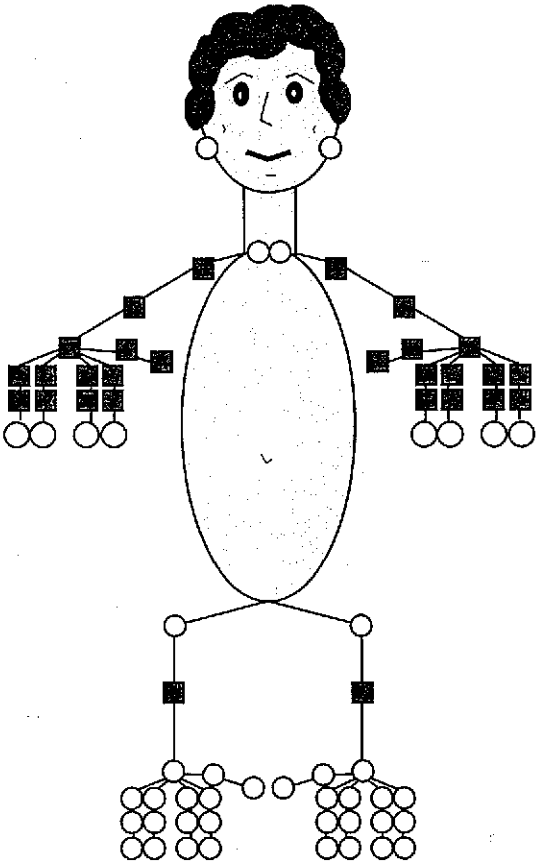

OTEČENI ZGLOBOVI
(br. oz.):

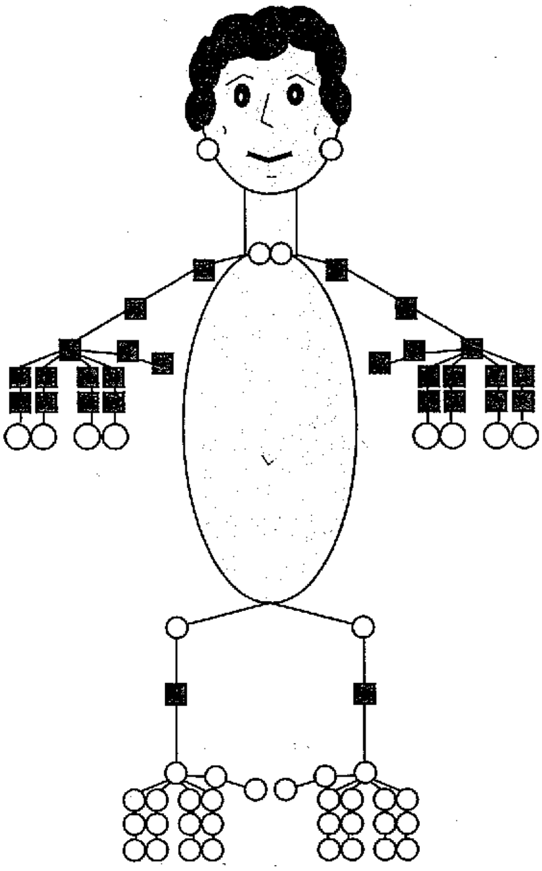

Sedimentacija eritrocita CRP nakon 1 h (mm):

Bolesnikova procjena aktivnosti bolesti (VAS; mm):

0	50	100
Nema aktivnosti		Najjača moguća aktivnost

DAS28 = $0,56 \times \sqrt{br.zbd.} + 0,28 \times \sqrt{br.oz.} + 0,7 \times \ln(SE) + 0,0142 \times VAS$

Enbrel
etanercept
