# Supplementary material for: The Influence of Rheumatoid Arthritis and Osteoarthritis on the Occurrence of Arterial Hypertension: An 8-Year Prospective Clinical Observational Cohort Study
Source: J Clin Med. 2023 Nov 18;12(22):7158. doi: 10.3390/jcm12227158 (PMC10672072; doi:10.3390/jcm12227158)
Supplement: Supplementary file 1 [file jcm-12-07158-s001.zip › S2 CRF OA initial visit.docx]

**UPITNIK ZA OSTEOARTRITIS ARTRITIS**

**Šifra/broj ID:_____**

**MBO:**

Ime:_____________________

Prezime:__________________

Godina rođenja:_______g

Adresa stanovanja:__________________________

Stručna sprema: NSS SSS VšSS VSS

Spol: M Ž

**OSTEOARTRITIS ARTRITIS:**

Kada je postavljena dijagnoza osteoartritisa (mjesec i godina): ___mj, ______g.

Da li ste do sada uzimali glukokortikoide, Medrol ili Decortin: DA NE

Koliko st dugo uzimali glukokotrikoide (mjeseci): _____mj.

Kolika je najviša dnevna doza glukokotrikoida koju ste uzimali: Decortin____mg

Medrol_____mg

Da li sada uzimate glukokortikoide, Medrol ili Decortin: DA NE

Koje lijekove sada uzima u liječenju reumatoidnog artritisa:

| **VRSTA LIJEKA** | **NAZIV LIJEKA** | **DNEVNA DOZA** |
| --- | --- | --- |
| NSAR |  |  |
| Analgetici |  |  |
| Drugi |  |  |
|  |  |  |
|  |  |  |
|  |  |  |
|  |  |  |
|  |  |  |
|  |  |  |
|  |  |  |
|  |  |  |
|  |  |  |
|  |  |  |

**KRVNI TLAK:**

Da li imate povišeni krvni tlak: DA NE

Kada je prvi put liječnik postavio dijagnozu povišenog krvnog tlaka (mjesec i godina):_____mj, ______g

Kolika je tada bila izmjerena vrijednost krvnog tlaka:_____/_____ mm Hg.

Kolika je do sada najviša izmjerena vrijednost krvnog tlaka:_____/_____ mm Hg.

Da li uzimate lijek koji snižava visoki krvni tlak: DA NE

Koje lijekove sada uzima u liječenju visokog krvnog tlaka:

| **VRSTA LIJEKA** | **NAZIV LIJEKA** | **DNEVNA DOZA** |
| --- | --- | --- |
| Betablokator |  |  |
| Alfablokator |  |  |
| ACE inhibitor |  |  |
| Blokator Ca kanala |  |  |
| ARB (AT1 blokator) |  |  |
| Diuretik |  |  |
| Drugi |  |  |
|  |  |  |
|  |  |  |
|  |  |  |

**MASNOĆE U KRVI:**

Da li imate povišene masnoće u krvi: DA NE

Da li uzimate lijek koji snižava masnoće u krvi: DA NE

**PUŠENJE:**

Jeste li ikad pušili: DA NE

Ako jeste, koliko ste ukupno godina pušili:________g.

Da li sada pušite: DA NE

Ako pušite, koliko cigareta dnevno pušite:________cigareta/dan.

**ŠEĆERNA BOLEST:**

Da li bolujete od šećerne bolesti: DA NE

Ako bolujete, kako se liječite: samo dijeta tablete inzulin inzulin + tablete

**PREHRANA:**

Jedete li voće ili pijete prirodni voćni sok svako dan: DA NE +1

Jedete li voće 2 puta dnevno: DA NE +1

Jedete li svježe ili kuhano povrće redovito jendom na dan: DA NE +1

Jedete li svježe ili kuhano povrće redovito više od jendom na dan: DA NE +1

Jedete li ribu redovito, najmanje 2 puta tjedno: DA NE +1

Jedete li više od jednom tjedno slaninu, pršut, kulen i sl: DA NE -1

Jedete li rižu ili tjesteninu 5 ili više puta tjedno: DA NE +1

Jedete li žitarice, crni kruh ili kruh sa cijelim zrnima za doručak: DA NE +1

Jedete li orašaste plodove najmanje 2 puta tjedno: DA NE +1

Upotrebljavate li maslinovo ulje za pripremu ili začin hrane: DA NE +1

Preskačete li doručak: DA NE -1

Jedete li mliječne proizvode za doručak: DA NE +1

Jedete li kupovni bijeli kruh i/ili peciva za doručak: DA NE -1

Jedete li najmanje 2 jogurta i/ili 40g sira dnevno: DA NE +1

Jedete li slatkiše nekoliko puta dnevno: DA NE -1

Pijete li crno vino svakodnevno do četvrtine litre: DA NE +1

Pijete li crno vino svakodnevno više od četvrtine litre: DA NE +1

Ukupan broj bodova (odgovor DA je + ili – jedan bod):__________

**BOLESTI SRCA:**

Da li ste se do sata liječili zbog srčanih bolesti: DA NE

Dijagnoze:____________________________

____________________________

____________________________

Ako jeste: Da li ste imali/imate zaduhu zbog slabosti srca: DA NE

Da li su vam oticale noge zbog slabosti srca: DA NE

Da li ste imali bolove u prsima (angina pect. Ili ekviv): DA NE

Da li ste imali/imate srčane artimije: DA NE

Koje artimije:_________________________________________

Antiaritmici: DA NE

Ako je odgovor DA, koji aniaritmici:

| GENERIČKI NAZIV | TVORNIČKI NAZIV | DNEVNA DOZA |
| --- | --- | --- |
|  |  |  |
|  |  |  |
|  |  |  |
|  |  |  |
|  |  |  |
|  |  |  |
|  |  |  |

Da li uzimate još neke lijekove: DA NE

Ako je odgovor DA, koje:

| GENERIČKI NAZIV | TVORNIČKI NAZIV | DNEVNA DOZA |
| --- | --- | --- |
|  |  |  |
|  |  |  |
|  |  |  |
|  |  |  |
|  |  |  |
|  |  |  |
|  |  |  |
|  |  |  |

**PREGLED:**

Tjelesna visina:_____cm

Tjelesna masa:_____kg

BMI:_____kg/m^2^

Struk:_____cm (mjeri se 1cm iznad criste iliace)

Bokovi:_____cm (mjeri se kao najširi opseg u području velikog tronhantera)

Omjer srtuk/bokovi:_____

Krvni tlak: (mjeriti u razmaku os 5 minuta)

1. Mjerenje:_____/_____ mm Hg.
2. Mjerenje:_____/_____ mm Hg.
3. Mjerenje:_____/_____ mm Hg.

Srednja vrijednost:_____/_____ mm Hg.

HAQ:_____

GH:_____

VAS:_____

Laboratorijski nalazi:

| **SE** | **CRP** | **RF** | **CCP** | **kolesterol** | **trigliceridi** | **LDL** | **HDL** | **kreatinin** | **GUK** |
| --- | --- | --- | --- | --- | --- | --- | --- | --- | --- |
|  |  |  |  |  |  |  |  |  |  |
|  |  |  |  |  |  |  |  |  |  |

| **OGTT (GUK 6.1-7.0 mmol/l)** |
| --- |
|  |

EKG – HLV: DA NE

FA: DA NE

OSTALI: DA NE

Ako je odgovor DA, koji:________________________________________________________
