# Supplementary material for: The Influence of Rheumatoid Arthritis and Osteoarthritis on the Occurrence of Arterial Hypertension: An 8-Year Prospective Clinical Observational Cohort Study
Source: J Clin Med. 2023 Nov 18;12(22):7158. doi: 10.3390/jcm12227158 (PMC10672072; doi:10.3390/jcm12227158)
Supplement: Supplementary file 1 [file jcm-12-07158-s001.zip › S3 CRF RA anual visit form.docx]

OBRAZAC ZA GODIŠNJE VIZITE RA:

Šifra/broj ID:_____

VIZITA BROJ:

IME I PREZIME:

MBO:

DATUM:

SIMPTOMI I NALAZI:

DIJAGNOZE (sve):

KVB (priložit nalaze ako je dijagnosticirana):

KVB RIZIČNI FAKTORI (označi ako postoje):

- HIPERTENZIJA
- ŠEĆERNA BOLEST
- DISLIPIDEMIJA
- PUŠENJE CIGARETA (promjene statusa)

LIJEKOVI (promjene):

EGZACERBACIJA (FLARE) OD POSLJEDNJE VIZITE (ako je odgovor da priložiti nalaze):

DRUGE UPALNE BOLESTI (ako ih ima priložiti nalaze):

KIRUŠKI ZAHVATI RADI RA (ako ih ima priložiti nalaze):

AKTIVNOST BOLESTI (DAS 28 CRP SCORE):
