# Supplementary material for: The Influence of Rheumatoid Arthritis and Osteoarthritis on the Occurrence of Arterial Hypertension: An 8-Year Prospective Clinical Observational Cohort Study
Source: J Clin Med. 2023 Nov 18;12(22):7158. doi: 10.3390/jcm12227158 (PMC10672072; doi:10.3390/jcm12227158)
Supplement: Supplementary file 1 [file jcm-12-07158-s001.zip › S4 CRF RA final visit.docx]

**Obrazac za bolesnike s RA Završna vizita**

**2008.g. ispunjavali ste prvi upitnik o Vašoj bolesti. To smo u ovom kontrolnom upitniku označili kao 1. pregled. Obavljali ste i godišnje vizire označene brojevima. Sada ispunjavate upitnik za kontrolni pregled koji će pokazati Vaše zdravstveno stanje kroz proteklih sedam godina, neki podaci se ponavljaju radi provjere.**

**Šifra/broj ID:_____**

**RA** (**označiti ✕**): seropozitivni ☐ **RA** (**označiti ✕**): erozivni ☐

seronegativni ☐ nepoznato ☐ neerozivni ☐ nepoznato ☐

Ime i prezime Datum pregleda

__________________ ___ ___ ______

Datum rođenja

________________________

Adresa i grad stanovanja: Kontakt telefon:

_______________________________ ______________________________

JMBG OIB  **MBO(Matični broj osiguranika)**

**___________________ ___________________ _____________________**

**ANAMNEZA**

REUMATOIDNI ARTRITIS:

Da li se od 1.pregleda Vaše stanje obzirom na osnovnu bolest (reumatoidni artritis) poboljšalo ili pogoršalo (zaokruži na slici)?

|  |  |  |  |  |  |
| --- | --- | --- | --- | --- | --- |
|  |  |  |  |  |  |

Izuzetno značajno blago jednako blago značajno izvrsno

pogoršano pogoršano pogoršano poboljšano poboljšano

Ukupno trajanje bolesti (RA): ______g.

Da li ste od 1. pregleda uzimali glukokortikoide:Medrol ili

Decortin(zaokružiti)? da ne

Ako je odgovor DA, odgovorite na slijedeća pitanja

Da li sada uzimate glukokortikoide: Medrol ili Decortin

(zaokružiti)? da ne

Koliko dugo ste od 1. pregleda ukupno uzimali Medrol ili Decortin

(navesti brojku u mjesecima)? ____mj.

Za bolesnike koji **sada** uzimaju GK(ispunjava liječnik, označiti X):

1.Korištenje glukokortikoida: **<** 3 mjeseca ☐ 3-6 mjeseci ☐

6 mjeseci -12mjeseci ☐ > 12 mjeseci ☐

2.Korištenje glukokortikoida**: prosječno** <7,5mg prednizona ili ekvivalent ☐

**prosječno** 7,5mg -20mg prednizona ili ekvivalent ☐

**prosječno** ≥20mg prednizona ili ekvivalent ☐

Za bolesnike koji **su uzimali** GK od 1. pregleda, ali ih **sada ne uzimaju** (ispunjava liječnik, označiti X):

1.Korištenje glukokortikoida: **<** 3 mjeseca ☐ 3-6 mjeseci ☐

6 mjeseci -12mjeseci ☐ > 12 mjeseci ☐

2.Korištenje glukokortikoida**: prosječno** <7,5mg prednizona ili ekvivalent ☐

**prosječno** 7,5mg -20mg prednizona ili ekvivalent ☐

**prosječno** ≥20mg prednizona ili ekvivalent ☐

**KRVNI TLAK:**

**Da li** vam je od 1.pregleda liječnik postavio dijagnozu povišenog krvnog tlaka

(zaokruži)? da ne

Ako je odgovor DA odgovorite na slijedeća pitanja:

**Kad** je prvi put liječnik postavio dijagnozu povišenog krvnog tlaka?

(upišite mjesec i godinu, npr. 06.2012.g.) __ mj. ____g.

Uzimate li lijek koji snižava visoki krvni tlak(zaokruži)? da ne

**MASNOĆE U KRVI:**

Da li imate povišene masnoće u krvi(zaokruži)? da ne

Da li uzimate lijek koji snižava masnoće u krvi? da ne

**PUŠENJE:**

Da li sada pušite (zaokružiti)? da ne

Da li ste bivši pušač (zaokružiti)? da ne

Koliko ste ukupno godina tijekom života pušili? ____ godina

Koliko ste prosječno cigareta dnevno pušili)? ____ cigareta

Molim izračunati broj kutija/godina odnosno pack/years na slijedeći način:

(broj cigareta na dan/20)x broj godina pušenja):_________

**ŠEĆERNA BOLEST:**

Da li bolujete od šećerne bolesti(zaokruži)? da ne

Ako je odgovor DA, da li uzimate inzulin ili tablete(zaokruži)? inzulin tablete

Da li je bolesnik **od 1.pregleda** liječen zbog bolesti srca? da ne

Ako je odgovor DA, napišite dijagnozu: _____________

_____________

KARDIOVASKULARNI DOGAĐAJI **OD 1.PREGLEDA**

(zaokružiti da ili ne, **ako je odgovor da, navesti i godinu događaja, np. 2012.g.)**

Da li je ispitaniku od 1. pregleda utvrđeno srčano popuštanje(dokumentirano): da (_______g.) ne

Da li je od 1. pregleda u ispitanika utvrđena periferna vaskularna bolest(arterijska insuficijencija) da (______g.) ne

Da li je od 1. pregleda u ispitanika utvrđena tranzitorna ishemijska ataka (TIA): da (_____g.) ne

Da li je od 1. pregleda u ispitanika utvrđen moždani udar? da (_____g.) ne

Da li je od 1. pregleda ispitanik prebolio srčani infarkt? da (_____g.) ne

Da li je od 1. pregleda u ispitanika utvrđena koronarna bolest (bez preboljelog infarkta, npr. implantacija stenta)? da (______g.) ne

Da li je od 1. pregleda u ispitanika utvrđena angina pektoris(dokumentirano)? da(______g.) ne

Da li je od 1. pregleda u ispitanika utvrđena aneurizma aorte? da (_______g.) ne

**POREMEĆAJI SRČANOG RITMA**

Da li je bolesnik od 1. pregleda imao aritmiju? da ne

Ako je odgovor DA , napišite koju aritmiju: ________________

Da li bolesnik uzima antiaritmike? da ne

Ako je odgovor DA, navedite koje:

generički naziv tvornički naziv dnevna doza

|  |  |  |
| --- | --- | --- |
|  |  |  |
|  |  |  |

**KOMPLIKACIJE:**

Da li je ispitanik od 1. pregleda liječen biološkom terapijom (zaokruži)? da ne

Da li je ispitanik sada na biološkoj terapiji(zaokruži)? da ne

Da li je ispitanik od 1. pregleda bio na biološkoj terapiji,

ali trenutno nije na biološkoj terapiji (zaokruži) da ne

| Da li je ispitaniku (**za sve bolesnike, neovisno o biološkoj terapiji**)  od 1. pregleda utvrđena: | Ako je utvrđena  komplikacija molim  navesti godinu,  npr. 2012. |
| --- | --- |

-teška infekcija (sepsa, infekcija koja zahtjeva hospitalizaciju) da ne _______g.

-tuberkuloza da ne _______g.

ako je odgovor da, molim zaokružiti: plućna ili izvan plućna

-reaktivacija hepatitisa B da ne _______g.

-reaktivacija hepatitisa C da ne _______g.

-ostale infekcije (isključujući akutni resp. infekt gornjih dišnih puteva) da ne _______g.

-citopenija (netropenija, pancitopenija, aplastična anemija) da ne _______g.

-demijelinizacijska bolest da ne _______g.

-plućna fibroza/intersticijska bolest pluća da ne _______g.

-granulomatozna bolest pluća da ne ______g.

**-zloćudna bolest**  da ne

*ako je odgovor da, molim zaokružiti vrstu zloćudne bolesti (broj);

melanom 1, nemelanomski rak kože 2, solidni tumor 3, limfom 4: 1 2 3 4

*u slučaju solidnog tumora upisati vrstu tumora ako je poznata ________________

*drugi oblik maligne bolesti ako nije uključen u prethodne skupine _____________

*godina kada je utvrđena zloćudna bolest ____________g.

**LIJEKOVI**

Koje lijekove bolesnik **sada** uzima za liječenje reumatoidnog artritisa (**zaokružiti lijek**)?

VRSTA LIJEKA

|  | Neselektivni NSAR |  |
| --- | --- | --- |
|  | Selektivni NSAR |  |
|  | jednostavni analgetik |  |
|  | opioidi |  |
|  |  |  |
|  | Kombinacija opioida/jedn.analgetika |  |
|  | Glukokortikoidi |  |
|  |  |  |
|  |  |  |
|  |  | Upisati ispod trajanje liječenja; broj godina, 1/2, 1, 1,5...) |
|  | Antimalarik |  |
|  | Metotreksat |  |
|  | Leflunomid |  |
|  | Sulfasalazin |  |
|  | Tofacitinib |  |
|  |  |  |
| **TNF alfa inhibitor(zaokružiti)** | da ne |  |
|  | Adalimumab |  |
|  | Infliksimab (Remicade) |  |
|  | Infliximab (Remsima) |  |
|  | Infliximab (Inflectra) |  |
|  | Etanercept |  |
|  | Golimumab |  |
|  | Certolizumab |  |
|  |  |  |
|  | Rituksimab |  |
|  | Tocilizumab |  |
|  | Abatacept |  |
|  | Anakinra |  |
|  | Drugo |  |
|  |  |  |
|  |  |  |

Koje je lijekove bolesnik **uzimao za liječenje RA od 1. pregleda, ali ih sada ne uzima**?

| VRSTA LIJEKA  (zaokružiti) | Trajanje liječenja (broj godina) : 1/2, 1, 1,5 | Vremenski period liječenja  (npr.2012-2013... | Uzrok prekida liječenja biološkim lijekom (upisati broj):  infuz.reakcija ili teža  reakcija na mjestu uboda 1  neučinkovitost 2  nuspojave 3  dugotrajna remisija 4 | Ako je uzrok prekida liječenja biološkim lijekom nuspojava ili komplikacija,molim navesti koja nuspojava | Ako je bolesnik liječen s više od jednim biološkim lijekom  molim unijeti redoslijed liječenja  (1,2,3..) |
| --- | --- | --- | --- | --- | --- |
| **Tofacitinib** |  |  |  |  |  |
| **TNF alfa inhibitor**  (zaokružiti)  da ne |  |  |  |  |  |
| Adalimumab |  |  |  |  |  |
| Infliksimab (Remicade) |  |  |  |  |  |
| Infliximab (Remsima) |  |  |  |  |  |
| Infliximab (Inflectra) |  |  |  |  |  |
| Etanercept |  |  |  |  |  |
| Golimumab |  |  |  |  |  |
| Certolizumab |  |  |  |  |  |
|  |  |  |  |  |  |
| **Rituksimab** |  |  |  |  |  |
| **Tocilizumab** |  |  |  |  |  |
| **Abatacept** |  |  |  |  |  |
| **Anakinra** |  |  |  |  |  |
| Drugo |  |  |  |  |  |
|  |  |  |  |  |  |
| **Metotreksat** |  |  |  |  |  |
| **Antimalarik** |  |  |  |  |  |
| **Leflunomid** |  |  |  |  |  |
| **Sulfasalazin** |  |  |  |  |  |

Koji bolesnik sada uzima lijek za snižavanje visokog krvnog tlaka?

Naziv lijeka VRSTA LIJEKA (zaokružiti)

|  | Betablokator |
| --- | --- |
|  | Alfablokator |
|  | ACE inhibitor |
|  | Inhibitor CA kanala |
|  | Inhibitor angioten.rec. |
|  | Diuretik |
|  | Drugi |
|  |  |

Da li bolesnik uzima neke druge lijekove(zaokruži)? da ne

Ako je odgovor DA, navedite koje:

1._______________

2._______________

3.______________

**Pregled**:

Arterijski tlak:3 mjerenja u mirovanju u razmaku od 5 minuta

1.mjerenje: ____/____ mmHg

2.mjerenje: ____/____ mmHg

3.mjerenje: ____/____ mmHg

Srednja vrijednost: ____/____ mmHg

Tjelesna visina:____cm

Tjelesna težina:____kg

BMI: ____

Struk *: ____ cm

Bokovi:* ____ cm

Omjer struka

i bokova: ____

*opseg struka se mjeri u ravnini 1cm iznad criste iliace

*opseg bokova se mjeri kao najširi opseg bokova u području velikog trohantera

DAS 28 CRP:____

HAQ: ____

GH (bolesnikova procjena općeg zdravlja 0-100): ____

VAS boli (0-10) ____

**Laboratorijski nalazi:**

| RF | Anti CCP | SE | Kolesterol | Trigl. | HDL | LDL | CRP | Kreatinin | HbA1c | GUK |
| --- | --- | --- | --- | --- | --- | --- | --- | --- | --- | --- |
|  |  |  |  |  |  |  |  |  |  |  |

Ako je GUK 6,1- 7.0 mmol/L

| OGTT |
| --- |
|  |

Ukoliko su dostupni podaci molim unijeti CRP i DAS 28 CRP u tablicu

| **Godina** | **CRP** | **DAS28 CRP** |
| --- | --- | --- |
| **2010.** |  |  |
| **2011.** |  |  |
| **2012.** |  |  |
| **2013.** |  |  |
| **2014.** |  |  |
| **2015.** |  |  |
| **2016.(samo ako je trenutni pregled 2017.g.** |  |  |

**Pretrage:**

EKG; molim priložiti EKG traku i zaokružiti:

HLK: da ne

FA: da ne

SRČANA FREKVENCIJA: _________/min

OSTALI POREMEĆAJI U EKG-u: da ne

Ako je odgovor DA, navedite poremećaj u EKG-u:

________________

**Metabolički sindrom** (ispuniti će se naknadno): da ne

Primjedbe (navedite dodatne podatke važne za stanje bolesnika):
