# Supplementary material for: The Influence of Rheumatoid Arthritis and Osteoarthritis on the Occurrence of Arterial Hypertension: An 8-Year Prospective Clinical Observational Cohort Study
Source: J Clin Med. 2023 Nov 18;12(22):7158. doi: 10.3390/jcm12227158 (PMC10672072; doi:10.3390/jcm12227158)
Supplement: Supplementary file 1 [file jcm-12-07158-s001.zip › S6 CRF OA final visit.docx]

**Obrazac za bolesnike s OA Završna vizita**

**2008.g. ispunjavali ste prvi upitnik o Vašoj bolesti. To smo u ovom kontrolnom upitniku označili kao 1. pregled. Sada ispunjavate upitnik za kontrolni pregled koji će pokazati Vaše zdravstveno stanje kroz proteklih sedam godina.**

**Šifra/broj ID:_____**

**Osteoartritis (liječnik ispunjava, označiti ✕ zahvaćene lokalizacije, moguće su kombinacije): ŠAKA ☐**

**KOLJENA ☐**

**KUKOVA ☐**

Ime i prezime Datum pregleda

__________________ ___ ___ ______

Adresa i mjesto stanovanja: Kontakt telefon:

_______________________________ ______________________________

Datum rođenja: ______________________

JMBG OIB  **MBO(Matični broj osiguranika)**

**___________________ ___________________ _____________________**

**ANAMNEZA**

**OSTEOARTRITIS:**

Da li je od 1.pregleda Vaše stanje obzirom na osnovnu bolest (RA) poboljšano, jednako ili pogoršano (zaokruži na slici)?

|  |  |  |  |  |  |
| --- | --- | --- | --- | --- | --- |
|  |  |  |  |  |  |

Izuzetno značajno blago jednako blago značajno izvrsno

pogoršano pogoršano pogoršano poboljšano poboljšano

Ukupno trajanje bolesti (OA): ______g.

Da li ste od prvog pregleda operirani radi osnovne bolesti (OA) ? (zaokruži) da ne

Oblik operacije (zaokruži): TEP KUKA PEP KUKA TEP KOLJENA

DRUGO (nadopuni)________________

**KRVNI TLAK:**

**Da li** vam je od 1.pregleda liječnik postavio dijagnozu povišenog krvnog tlaka

(zaokruži)? da ne

Ako je odgovor DA odgovorite na slijedeća pitanja:

**Kad** je prvi put liječnik postavio dijagnozu povišenog krvnog tlaka?

(upišite mjesec i godinu, npr. 06.2012.g.) __ mj. ____g.

Da li uzimate lijek koji snižava visoki krvni tlak(zaokruži)? da ne

**MASNOĆE U KRVI:**

Da li imate povišene masnoće u krvi(zaokruži)? da ne

Da li uzimate lijek koji snižava masnoće u krvi? da ne

**PUŠENJE:**

Da li sada pušite (zaokružiti)? da ne

Da li ste bivši pušač (zaokružiti)? da ne

Koliko ste ukupno godina tijekom života pušili? ____ godina

Koliko ste prosječno cigareta dnevno pušili)? ____ cigareta

Molim izračunati broj kutija/godina odnosno pack/years na slijedeći način:

(broj cigareta na dan/20)x broj godina pušenja):_________

ŠEĆERNA BOLEST:

Da li bolujete od šećerne bolesti(zaokruži)? da ne

Ako je odgovor DA, da li uzimate inzulin ili tablete(zaokruži)? inzulin tablete

Da li je bolesnik **od 1.pregleda** liječen zbog bolesti srca? da ne

Ako je odgovor DA, napišite dijagnozu: _____________

_____________

Koje lijekove bolesnik sada uzima za liječenje osteoartritisa (zaokružiti)?

VRSTA LIJEKA DNEVNA DOZA

| jednostavni analgetik |  |
| --- | --- |
| Neselektivni NSAR |  |
| Selektivni NSAR |  |
| Opioidi |  |
| Kombinacija analgetika/opioida |  |
| Drugo |  |
|  |  |

Koji bolesnik sada uzima lijek za snižavanje visokog krvnog tlaka(zaokružiti)?

NAZIV LIJEKA VRSTA LIJEKA

|  | Betablokator |
| --- | --- |
|  | Alfablokator |
|  | ACE inhibitor |
|  | Inhibitor CA kanala |
|  | Inhibitor angioten.rec. |
|  | Diuretik |
|  | Drugi |
|  |  |

KARDIOVAKULARNI DOGAĐAJI (zaokružiti da ili ne, **ako je odgovor da, navesti i godinu događaja, np. 2012.g.)**

Da li je ispitaniku od 1. pregleda utvrđeno srčano popuštanje(dokumentirano): da (___g.) ne

Da li je od 1. pregleda u ispitanika utvrđena periferna vaskularna bolest(arterijska insuficijencija) da (___g.) ne

Da li je od 1. pregleda u ispitanika utvrđena tranzitorna ishemijska ataka (TIA):

da (___g.) ne

Da li je od 1. pregleda u ispitanika utvrđen moždani udar? da (___g.) ne

Da li je od 1. pregleda ispitanik prebolio srčani infarkt? da (___g.) ne

Da li je od 1. pregleda u ispitanika utvrđena koronarna bolest (bez preboljelog infarkta, npr. implantacija stenta)? da (___g.) ne

Da li je od 1. pregleda u ispitanika utvrđena angina pektoris(dokumentirano)? da(___g.) ne

Da li je od 1. pregleda u ispitanika utvrđena aneurizma aorte? da (___g.) ne

**POREMEĆAJI RITMA**

Da li je bolesnik od 1. pregleda imao aritmiju? da ne

Ako je odgovor DA , napišite koju aritmiju: ________________

Da li bolesnik uzima antiaritmike? da ne

Ako je odgovor Da, navedite koje:

generički naziv tvornički naziv dnevna doza

|  |  |  |
| --- | --- | --- |
|  |  |  |

Da li je bolesnik od 1. pregleda imao aritmiju? da ne

Ako je odgovor DA , napišite koju aritmiju: ________________

Da li bolesnik uzima antiaritmike? da ne

Ako je odgovor DA, navedite koje:

generički naziv tvornički naziv dnevna doza

|  |  |  |
| --- | --- | --- |
|  |  |  |
|  |  |  |

**KOMPLIKACIJE:**

| Da li je ispitaniku  od 1. pregleda utvrđena: | Ako je utvrđena  komplikacija molim  navesti godinu,  npr. 2012. |
| --- | --- |

-teška infekcija (sepsa, infekcija koja zahtjeva hospitalizaciju) da ne _______g.

-tuberkuloza da ne _______g.

ako je odgovor da, molim zaokružiti: plućna ili izvan plućna

-reaktivacija hepatitisa B da ne _______g.

-reaktivacija hepatitisa C da ne _______g.

-ostale infekcije (isključujući akutni resp. infekt gornjih dišnih puteva) da ne _______g.

-citopenija (netropenija, pancitopenija, aplastična anemija) da ne _______g.

-demijelinizacijska bolest da ne _______g.

-plućna fibroza/intersticijska bolest pluća da ne _______g.

-granulomatozna bolest pluća da ne ______g.

**-zloćudna bolest**  da ne

*ako je odgovor da, molim zaokružiti vrstu zloćudne bolesti (broj);

melanom 1, nemelanomski rak kože 2, solidni tumor 3, limfom 4: 1 2 3 4

*u slučaju solidnog tumora upisati vrstu tumora ako je poznata ________________

*drugi oblik maligne bolesti ako nije uključen u prethodne skupine _____________

*godina kada je utvrđena zloćudna bolest ____________g.

Da li bolesnik uzima neke druge lijekove(zaokruži)? da ne

Ako DA, koje(navedite naziv)?

1._______________ 3._______________

2._______________ 4._______________

**Pregled**:

Arterijski tlak:3 mjerenja u mirovanju u razmaku od 5 minuta

1.mjerenje: ____/____ mmHg

2.mjerenje: ____/____ mmHg

3.mjerenje: ____/____ mmHg

Srednja vrijednost: ____/____ mmHg

Tjelesna visina:____cm

Tjelesna težina:____kg

BMI: ____

Struk *: ____ cm

Bokovi *: ____ cm

Omjer struka i bokova: ____

*opseg struka se mjeri u ravnini 1cm iznad criste iliace

*opseg bokova se mjeri kao najširi opseg bokova u području velikog trohantera

Funkcijski testovi:

HAQ: ____

GH (bolesnikova procjena općeg zdravlja 0-100): ____

VAS boli (0-10) ____

**ZAHVAĆENOST ZGLOBOVA:**

**1.molim zaokružiti zahvaćene zglobove:**

LIJEVA ŠAKA: 1 zglob (1bod) 2 zgloba (1 bod) 3 i više zglobova (2 boda)

DESNA ŠAKA: 1 zglob (1bod) 2 zgloba (1 bod) 3 i više zglobova (2 boda)

KUK LIJEVI (2 boda) KUK DESNI (2 boda)

KOLJENO LIJEVO (2 boda) KOLJENO DESNO (2 boda)

**2.ukupno broj zahvaćenih zgloba (zbroj bodova iz zagrada):________**

**3.Težina OA koljena (iz upitnika u prilogu):**

**Težina OA kuka (iz upitnika u prilogu):**

**Težina OA šaka (iz upitnika u prilogu):**

**Laboratorijski nalazi:**

| SE | Kolesterol | Trigl. | HDL | LDL | CRP | Kreatinin | HbA1c | GUK |
| --- | --- | --- | --- | --- | --- | --- | --- | --- |
|  |  |  |  |  |  |  |  |  |

Ako je GUK 6,1- 7.0 mmol/L

| OGTT |
| --- |
|  |

**Pretrage:**

EKG; molim priložiti EKG traku i zaokružiti:

HLK: da ne

FA: da ne

SRČANA FREKVENCIJA: _________/min

OSTALI POREMEĆAJI U EKG-u: da ne

Ako je odgovor DA, navedite poremećaj u EKG-u:

________________

**Metabolički sindrom (ispuniti će se naknadno): da ne**

Primjedbe (navedite dodatne podatke važne za stanje bolesnika):
