# Supplementary material for: The Influence of Rheumatoid Arthritis and Osteoarthritis on the Occurrence of Arterial Hypertension: An 8-Year Prospective Clinical Observational Cohort Study
Source: J Clin Med. 2023 Nov 18;12(22):7158. doi: 10.3390/jcm12227158 (PMC10672072; doi:10.3390/jcm12227158)
Supplement: Supplementary file 1 [file jcm-12-07158-s001.zip › S7 CRF OA final visit - unavailable.docx]

**Obrazac za bolesnike s OA**

**Šifra/broj ID:_____**

Ime i prezime

__________________

Adresa i mjesto stanovanja:

_______________________________

Datum rođenja: ______________________

JMBG _____________________

OIB ______________________

MBO(Matični broj osiguranika)__________________

**Zaokružiti razlog nedostupnosti ispitanika:**

1.nepotpuni kontakt podaci

2.ispitanik ne želi sudjelovati

3.smrtni ishod

4.drugo_______

Da li je od 1. pregleda nastupio smrtni ishod (zaokruži): da (_____g.) ne nepoznato

Ako je odgovor da, da li je uzrok smrti bio kardiovaskularni da ne

napišite uzrok smrti ukoliko je poznat: ____________

Poznati kardiovaskularni faktori rizika:
