# Supplementary material for: The Influence of Rheumatoid Arthritis and Osteoarthritis on the Occurrence of Arterial Hypertension: An 8-Year Prospective Clinical Observational Cohort Study
Source: J Clin Med. 2023 Nov 18;12(22):7158. doi: 10.3390/jcm12227158 (PMC10672072; doi:10.3390/jcm12227158)
Supplement: Supplementary file 1 [file jcm-12-07158-s001.zip › S10 LEQUESNE KNEE OSTEOARTRITIS.docx]

**LEQUESNE OSTEOARTRITIS KOLJENA**

| **BOL ILI NELAGODA** |  |
| --- | --- |
| *NOĆU* |  |
| Bez bolova ili zanemarivi bolovi | 0 |
| Samo kod pokreta ili nekoh položaja | 1 |
| U mirovanju | 2 |
| *UJUTRO NAKON USTAJANJA (ZAKOČENOST ILI BOLOVI)* |  |
| <1min | 0 |
| 1-15min | 1 |
| ≥15min | 2 |
| *NAKON 30 min STAJANJA* |  |
| Ne ili da | 0 ili 1 |
| ZA VRIJEME HODA |  |
| bezbolno | 0 |
| Bol nakon savladavanja neke udaljenosti | 1 |
| Bol od početka hoda uz pogoršanje tijekom hoda | 2 |
| *BOL ILI NELAGODA NAKON USTAJANJA IZ SJEDEĆEG POLOŽAJA BEZ POMOĆI RUKU* |  |
| ne ili da | 0 ili 1 |
| **MAKSIMALNA UDALJENOST PRI HODU (uz bolove)** |  |
| Neograničena | 0 |
| Veća od 1km, ali ograničena | 1 |
| Oko 1 km za 15 minuta | 2 |
| 500-900m za 8-15minuta | 3 |
| 300-500m | 4 |
| 100-300m | 5 |
| <100m | 6 |
| Uz pomoć jednog štapa ili štake | 1 |
| Uz pomoć 2 štapa ili 2 štake | 2 |
| **AKTIVNOSTI SVAKODNEVNOG ŽIVOTA** | 0-bez poteškoća  1-s poteškoćama  2-nesposoban |
| Čučanj |  |
| Hod pod neravnom |  |
| Hod uz stepenice |  |
| Hod niz stepenice |  |

**ZBROJ:**
