# Supplementary material for: The Influence of Rheumatoid Arthritis and Osteoarthritis on the Occurrence of Arterial Hypertension: An 8-Year Prospective Clinical Observational Cohort Study
Source: J Clin Med. 2023 Nov 18;12(22):7158. doi: 10.3390/jcm12227158 (PMC10672072; doi:10.3390/jcm12227158)
Supplement: Supplementary file 1 [file jcm-12-07158-s001.zip › S11 OSTEOARTHRITS FIST INDEX.docx]

AS INDEX OSTEOARTRITIS ŠAKE

**BOL**

1. Kako bi opisali bolove u zglobovima šaka u posljednja 2 dana (zaokruži)?

0 (bez bolova)

1 (slabi bolovi)

2 (značajni bolovi)

3 (jaki bolovi)

4 (izraziti bolovi) **broj bodova x 5=**

**UKOČENOST**

2.Kako bi opisali osjećaj ukočenosti u šakama u posljednja 2 dana (zaokruži)?

0 (bez ukočenosti)

1 (ukočenost do 15minuta)

2 (ukočenost 15 do 30minuta)

3 (ukočenosti 30minuta do 1 sat)

4 (ukočenosti > 1 sat) **broj bodova=**

| **FUNKCIJA** | 0 (mogu, bez poteškoća)  1 (mogu, uz manje poteškoće)  2 (mogu, otežano)  3 (mogu, uz velike poteškoće)  4 (ne mogu uopće) |
| --- | --- |
| 1.Da li možete okrenuti ključ u bravi? |  |
| 2.Da li možete rezati meso nožem? |  |
| 3.Da li možete rezati papir škarama? |  |
| 4.Da li možete uhvatiti bocu vode rukom? |  |
| 5.Da li možete stisnuti šaku? |  |
| 6.Da li možete zavezati čvor? |  |
| 7.Da li možete šivati/koristiti odvijač? |  |
| 8.Da li možete zakopčati dugmad? |  |
| 9.Da li možete pisati kroz duže vrijeme? |  |
| **broj bodova=** |  |

**UKUPAN BROJ BODOVA(iz sve 3 skupine pitanja, maksimalan zbroj 60):**
