# Supplementary material for: The Influence of Rheumatoid Arthritis and Osteoarthritis on the Occurrence of Arterial Hypertension: An 8-Year Prospective Clinical Observational Cohort Study
Source: J Clin Med. 2023 Nov 18;12(22):7158. doi: 10.3390/jcm12227158 (PMC10672072; doi:10.3390/jcm12227158)
Supplement: Supplementary file 1 [file jcm-12-07158-s001.zip › S1 CRF RA initial visit.doc]

**UPITNIK ZA RA**

**MBO:**

Ime i prezime	Datum

__________________	___ ___ ______

Adresa stanovanja

Stručna sprema (zaokruži)	NSS	SSS	VšSS	VSS

**ANAMNEZA(ispunjava ispitanik):**

Godina rođenja: ______g.

Spol(zaokruži):

muški	ženski

REUMATOIDNI ARTRITIS:

Kada je postavljena dijagnoza reumatoidnog artritisa

(navedite mjesec i godinu)? ___mj. _______g.

Da li ste do sada uzimali glukokortikoide:Medrol ili

Decortin(zaokružiti)? da	ne

Koliko dugo ste otprilike uzimali Medrol ili Decortin

(navesti brojku u mjesecima)? ____mj.

Kolika je najviša dnevna doza Medrola odnosno

Decortina koju ste do sada uzimali? Medrol _____mg

Decortin____ mg

Da li sada uzimate glukokortikoide: Medrol ili Decortin

(zaokružiti)? da	ne

KRVNI TLAK:

Da li imate povišeni krvni tlak (zaokruži)? da	ne	Kad je prvi put liječnik postavio dijagnozu povišenog krvnog tlaka

(upišite mjesec i godinu)? __ mj. ______g.

Kolika je tada bila izmjerena vrijednost krvnog tlaka? ___/___

Kolika je do sada najviša izmjerena vrijednost krvnog tlaka? ___/___

Da li uzimate lijek koji snižava visoki krvni tlak(zaokruži)? da	ne

MASNOĆE U KRVI:

Da li imate povišene masnoće u krvi(zaokruži)? da	ne

Da li uzimate lijek koji snižava masnoće u krvi? da	ne

PUŠENJE:

Jeste li ikad pušili (zaokruži)? da	ne

Ako je odgovor DA, koliko ste ukupno godina pušili? ____ godina

Da li sada pušite(zaokruži)? da	ne

Ako je odgovor DA, koliko cigareta dnevno pušite? _____cig./dan

ŠEĆERNA BOLEST:

Da li bolujete od šećerne bolesti (zaokruži)? da	ne

Ako je odgovor DA, da li uzimate inzulin ili tablete(zaokruži)? inzulin	tablete

PREHRANA(zaokruži):

| Jedete voće ili pijete prirodni voćni sok svaki dan? | da | ne | +1 |
| --- | --- | --- | --- |
| Jedete voće 2 puta dnevno? | da | ne | +1 |
| Jedete svježe ili kuhano povrće redovito jednom na dan? | da | ne | +1 |
| Jedete svježe ili kuhano povrće više od jednom na dan? | da | ne | +1 |
| Jedete ribu redovito, najmanje 2-3 puta tjedno? | da | ne | +1 |
| Više od 1 tjedno jedete slaninu, pršut, kulen? | da | ne | -1 |
| Konzumirate rižu ili tjesteninu skoro svaki dan, 5 ili više puta tjedno? | da | ne | +1 |
| Jedete žitarice (pahuljice, soja, smeđa riža..)  ili crni kruh, kruh sa cijelim zrnima za doručak? | da | ne | +1 |
| Redovito jedete orašaste plodove, najmanje 2-3 puta tjedno? | da | ne | +1 |
| Koristite maslinovo ulje kod pripreme ili začina hrane? | da | ne | +1 |
| Preskačete doručak? | da | ne | -1 |
| Jedete mliječne prozvode za doručak(jogurt, mlijeko..) | da | ne | +1 |
| Jedete kupovna bijeli kruh i peciva za doručak? | da | ne | -1 |
| Jedete 2 jogurta i/ili nekog sira (40g) dnevno? | da | ne | +1 |
| Jedete bombone i slatkiše nekoliko puta dnevno? | da | ne | -1 |
| Pijete li crno vino svakodnevno do četvrt litre? | da | ne | +1 |
| Pijete li crno vino svakodnevno više od četvrt litre? | da | ne | -1 |
| Ukupan broj bodova |  |  |  |

BOLESTI SRCA(zaokruži):

Da li ste se dosad liječili zbog srčanih tegoba? da	ne

Ako je odgovor DA, odgovorite na slijedeća pitanja:

Da li ste imali zaduhu zbog slabosti srca? da	ne

Da li su vam oticale noge zbog slabosti srca? da	ne

Da li ste imali bolove u prsima (angina pektoris)? da	ne

Da li ste imali aritmiju (poremećaj srčanog ritma)? da	ne

Ako znate, napišite dijagnozu bolesti srca zbog koje ste se liječili: ______________

______________

**ANAMNEZA(ispunjava liječnik).**

Koje lijekove bolesnik sada uzima za liječenje reumatoidnog artritisa?

NAZIV LIJEKA	VRSTA LIJEKA	DNEVNA DOZA

|  | Glukokortikoidi |  |
| --- | --- | --- |
|  | Metotreksat |  |
|  | Sulfasalazin |  |
|  | Adalimumab |  |
|  | Leflunonamid |  |
|  | Infliksimab |  |
|  | Rituksimab |  |
|  | NSAR |  |
|  | Analgetik |  |
|  | Drugi |  |

Koji bolesnik sada uzima lijek za snižavanje visokog krvnog tlaka?

NAZIV LIJEKA	VRSTA	LIJEKA	DNEVNA DOZA

|  | Betablokator |  |
| --- | --- | --- |
|  | Alfablokator |  |
|  | ACE inhibitor |  |
|  | Inhibitor CA kanala |  |
|  | Inhibitor angioten.rec. |  |
|  | Diuretik |  |
|  | Drugi |  |
|  |  |  |

Da li se bolesnik dosad liječio zbog bolesti srca? da	ne

Ako je odgovor DA, napišite dijagnozu: _____________

_____________

_____________

Da li je bolesnik dosad imao aritmiju? da	ne

Ako je odgovor DA, napišite koju aritmiju: ________________

________________

Da li bolesnik uzima antiaritmike? da	ne

Ako je odgovor DA ,navedite koje:

generički naziv	tvornički naziv	dnevna doza

Da li bolesnik uzima neke druge lijekove (zaokruži)? da	ne

Ako DA, koje(navedite naziv)?

1._______________

2._______________

3._______________

4._______________

5._______________

6._______________

**Pregled**:

Arterijski tlak:3 mjerenja u mirovanju u razmaku od 5 minuta

1.mjerenje: ____/____ mmHg

2.mjerenje: ____/____ mmHg

3.mjerenje: ____/____ mmHg

Srednja vrijednost: ____/____ mmHg

Tjelesna visina:____cm

Tjelesna težina:____kg

BMI: ____

Struk *: ____ cm

Bokovi:*	____ cm

Omjer struka

i bokova: ____

*opseg struka se mjeri u ravnini 1cm iznad criste iliace

*opseg bokova se mjeri kao najširi opseg bokova u području velikog trohantera

DAS 28 (CRP):____

HAQ: ____

GH: ____

VAS: _____

**Laboratorijski nalazi:**

| RF | CRP | SE | Kolest. | HDL | LDL | Trigl. |
| --- | --- | --- | --- | --- | --- | --- |
|  |  |  |  |  |  |  |

| Kreatinin | GUK | mikroalbuminurija |
| --- | --- | --- |
|  |  |  |

Ako je GUK 6,1- 7.0 mmol/L

OGTT

**Pretrage:**

EKG(zaokružite):

HLK: da	ne

FA: da	ne

OSTALI POREMEĆAJI U EKG-u: da	ne

Ako je odgovor DA, navedite poremećaj u EKG-u:

________________

________________

________________
