# Supplementary material for: The Influence of Rheumatoid Arthritis and Osteoarthritis on the Occurrence of Arterial Hypertension: An 8-Year Prospective Clinical Observational Cohort Study
Source: J Clin Med. 2023 Nov 18;12(22):7158. doi: 10.3390/jcm12227158 (PMC10672072; doi:10.3390/jcm12227158)
Supplement: Supplementary file 1 [file jcm-12-07158-s001.zip › S8 HAQ form.doc]

| UPITNIK ZA PROCJENU ZDRAVSTVENOG STANJA  HEALTH ASSESSMENT QUESTIONNAIRE - HAQ | | | | |  |  | | | | | | | |
| --- | --- | --- | --- | --- | --- | --- | --- | --- | --- | --- | --- | --- | --- |
| **Ime i prezime**__________________________________ **Datum**_____________________________________ | | | | | PATKEY#  QUESTDAT |  | | | | | | | |
| U ovom odjeljku želimo saznati kako bolest utječe na Vaše funkcioniranje u svakodnevnom životu. Ukoliko imate neke primjedbe, molimo vas da ih napišete na poleđini ove stranice.  **Označite (X) odgovor koji najbolje opisuje vaše uobičajene sposobnosti TIJEKOM PROTEKLIH TJEDAN DANA:** | | | | | HAQADMIN  QUESTYPE |  | |  | |  | |  | |
| **ODIJEVANJE I OSOBNA HIGIJENA**  Možete li: | Bez IKAKVIH  poteškoća | Uz ODREÐENE  poteškoće | Uz MNOGO  poteškoća | NE MOGU izvesti | PMSVIS  RASTUDY  QUESTNUM |  | | | | | | | |
| - Samostalno se odjenuti, vezati cipele i zakopčati dugmad? | _____ | _____ | _____ | _____ |  |  | | | | | | | |
| - Oprati kosu? | _____ | _____ | _____ | _____ | DRESSNEW |  | | | | | | | |
|  | | | | |  |  |  | |  | |  | |  |
| **USTAJANJE**  Možete li: |  |  |  |  |  |  | | | | | | | |
| - Ustati iz stolice? | _____ | _____ | _____ | _____ |  |  | | | | | | | |
| - Leći i ustati iz kreveta? | _____ | _____ | _____ | _____ | RISENEW |  | | | | | | | |
|  | | | | |  |  |  | |  | |  | |  |
| PREHRANA  Možete li: |  |  |  |  |  |  | | | | | | | |
| - Rezati meso? | _____ | _____ | _____ | _____ |  |  | | | | | | | |
| - Podići punu šalicu ili čašu do usta? | _____ | _____ | _____ | _____ |  |  | | | | | | | |
| - Otvoriti rukama novi tetrapak mlijeka? | _____ | _____ | _____ | _____ | EATNEW |  | | | | | | | |
|  | | | | |  |  |  | |  | |  | |  |
| HODANJE  Možete li: |  |  |  |  |  |  | | | | | | | |
| - Šetati izvan kuće po ravnom tlu? | _____ | _____ | _____ | _____ |  |  | | | | | | | |
| - Popeti se pet stepenica? | _____ | _____ | _____ | _____ | WALKNEW |  | | | | | | | |
|  |  |  |  |  |  |  | | | | | | | |
| Molimo označite (X) sva POMAGALA ILI NAPRAVE koje obično koristite za obavljanje gore navedenih aktivnosti:  _____ Štap _____ Pomagala za odijevanje (kuke za zakopčavanje dugmadi, žlica za obuvanje s dugom drškom itd.)  _____ Hodalica _____ Prilagođeni ili posebni kuhinjski pribor  _____ Štake _____ Posebne ili uzdignute stolice  _____ Invalidska kolica _____ Drugo (navedite:______________________) | | | | | DRSGASST  RISEASST |  | | | | | | | |
| **Molimo označite (X) djelatnosti za koje obično trebate POMOĆ DRUGE OSOBE:**  _____ Odijevanje i osobna higijena _____ Prehrana  _____ Ustajanje _____ Hodanje | | | | | EATASST  WALKASST |  | | | | | | | |

| **Označite (X) odgovor koji najbolje opisuje vaše uobičajene sposobnosti TIJEKOM PROTEKLIH TJEDAN DANA:** | | | | | | |  | |  |  |  |  |
| --- | --- | --- | --- | --- | --- | --- | --- | --- | --- | --- | --- | --- |
| HIGIJENA  Možete li: | | Bez IKAKVIH  poteškoća | Uz ODREÐENE  poteškoće | | Uz MNOGO  poteškoća | NE MOGU izvesti |  |  | | | | |
| - Prati i brisati tijelo? | | _____ | _____ | | _____ | _____ |  |  | | | | |
| - Kupati se sjedeći u kadi? | | _____ | _____ | | _____ | _____ |  |  | | | | |
| - Sjesti i ustati s nužnika? | | _____ | _____ | | _____ | _____ | HYGNNEW |  | | | | |
| DOHVAT  Možete li: | |  |  | |  |  |  |  | | | | |
| - Dohvatiti i skinuti predmet od 2 kg (na primjer jednu veću vrećicu šećera) s mjesta malo iznad vaše glave? | | _____ | _____ | | _____ | _____ |  |  | | | | |
| - Sagnuti se i podići odjeću s poda? | | _____ | _____ | | _____ | _____ | REACHNEW |  | | | | |
|  | |  |  | |  |  |  |  | | | | |
| STISAK  Možete li: | |  |  | |  |  |  |  | | | | |
| - Otvoriti vrata automobila? | | _____ | _____ | | _____ | _____ |  |  | | | | |
| - Otvoriti staklenku koja je već bila otvarana? | | _____ | _____ | | _____ | _____ |  |  | | | | |
| - Otvoriti i zatvoriti slavinu? | | _____ | _____ | | _____ | _____ | GRIPNEW |  | | | | |
| AKTIVNOSTI  Možete li: | |  |  | |  |  |  |  | | | | |
| - Izaći iz kuće u kupovinu i obaviti razne poslove? | | _____ | _____ | | _____ | _____ |  |  | | | | |
| - Ući i izaći iz automobila? | | _____ | _____ | | _____ | _____ |  |  | | | | |
| - Obaviti kućne poslove poput usisavanja prašine ili uređenja dvorišta? | | _____ | _____ | | _____ | _____ | ACTIVNEW |  | | | | |
| **Molimo označite (X) sva POMAGALA ILI NAPRAVE koje obično koristite za obavljanje gore navedenih aktivnosti:**  _____ Uzdignuta sjedalica na nužniku _____ Drška uz kadu  _____ Sjedalica u kadi _____ Pomagala s dugom drškom za dohvaćanje predmeta  _____ Otvarač za staklenke (za već _____ Pomagala s dugom drškom za dohvaćanje u otvarane staklenke) kupaonici (na primjer četka za tijelo)  _____ Drugo (navedite: ____________________) | | | | | | |  |  | | | | |
| **Molimo označite (X) djelatnosti za koje obično trebate POMOĆ DRUGE OSOBE:**  _____ Higijena _____ Uzimanje i otvaranje predmeta  _____ Dohvat _____ Izlasci i kućni poslovi | | | | | | | HYGNASST  RCHASST  GRIPASST  ACTVASST |  | | | | |
| Također želimo saznati imate li ili nemate bolove zbog svoje bolesti.  **Koliko ste jake bolove imali zbog svoje bolesti PROTEKLOG TJEDNA:**  Povucite okomitu (I) crtu na donjoj liniji kako biste označili jačinu tih bolova. | | | | | | |  |  | | | | |
| BEZ BOLOVA 0 |  | | | JAKI BOLOVI 100 | | | PAINSCAL |  | | | | |
|  |  | | |  |  |  |  |  | | | | |
